# Under-reporting of non-fatal occupational injuries among precarious and non-precarious workers in Sweden

**DOI:** 10.1136/oemed-2021-107856

**Published:** 2021-09-20

**Authors:** Bertina Kreshpaj, Theo Bodin, David H Wegman, Nuria Matilla-Santander, Bo Burstrom, Katarina Kjellberg, Letitia Davis, Tomas Hemmingsson, Johanna Jonsson, Carin Håkansta, Cecilia Orellana

**Affiliations:** 1 Unit of Occupational Medicine, Institute of Environmental Medicine, Karolinska Institutet, Stockholm, Sweden; 2 Stockholm County Council, Stockholm, Sweden; 3 University of Massachusetts Lowell, Lowell, Massachusetts, USA; 4 Department of Public Health Sciences, Karolinska Institutet, Stockholm, Sweden; 5 Researcher, Cambridge, Massachusetts, USA; 6 Department of Public Health Sciences, Stockholm University, Stockholm, Sweden; 7 Department of Working Life Science, Karlstad University, Karlstad, Sweden

**Keywords:** public health, epidemiology, occupational Health

## Abstract

**Background:**

Under-reporting of occupational injuries (OIs) among precariously employed workers in Sweden challenges effective surveillance of OIs and targeted preventive measures.

**Objective:**

To estimate the magnitude of under-reporting of OIs among precarious and non-precarious workers in Sweden in 2013.

**Methods:**

Capture–recapture methods were applied using the national OIs register and records from a labour market insurance company. Employed workers 18–65 resident in Sweden in 2013 were included in the study (n=82 949 OIs). Precarious employment was operationalised using the national labour market register, while injury severity was constructed from the National Patient Register. Under-reporting estimates were computed stratifying by OIs severity and by sociodemographic characteristics, occupations and precarious employment.

**Results:**

Under-reporting of OIs followed a dose–response pattern according to the levels of precariousness (the higher the precarious level, the higher the under-reporting) being for the precarious group (22.6%, 95% CI 21.3% to 23.8%), followed by the borderline precarious (17.6%, 95% CI 17.1% to 18.2%) and lastly the non-precarious (15.0%, 95% CI 14.7% to 15.3%). Under-reporting of OIs, decreased as the injury severity increased and was higher with highest level of precariousness in all groups of severity. We also observed higher under-reporting estimates among all occupations in the precarious and borderline precarious groups as compared with the non-precarious ones.

**Conclusions:**

This is the first register-based study to empirically demonstrate in Sweden that under-reporting of OIs is 50% higher among precariously employed workers. OIs under-reporting may represent unrecognised injuries that especially burden precariously employed workers as financial, health and social consequences shift from the employer to the employee.

**Key messages**

**What is already known about this subject?**

- A rich literature indicates a positive association between certain dimensions of precarious employment and occupational injuries (OIs), as well as substantial under-reporting. Qualitative studies have identified the main reasons why precarious workers may decide to not report an injury, but little is known about the extent of under-reporting in this group.

**What are the new findings**

- Under-reporting of OIs is 50% higher among precariously employed workers as compared with those in standard employment relationships.
- Across all sociodemographic characteristics, there is likely to be more under-reporting of OIs among precariously employed workers.
- Under-reporting remains highest among the most precarious groups irrespectively of injury severity.
- Individual dimensions of precarious employment were associated with highest under-reporting, such as income, workers with unstable employment and multiple job holders with more than three employers.

**How might this impact on policy or clinical practice in the foreseeable future?**

- By increasing the understanding between precarious employment and under-reporting of OIs, organisations may learn better approaches to improve reporting, address root causes of workplace injuries, and design health and safety programmes aimed at tackling specific component of the workforce—for example, precariously employed workers.

## Introduction

Under-reporting of occupational injuries (OIs), illnesses and other safety and health indicators negatively affect surveillance and targeted preventive measures.^1–3^ In the ‘90s, the under-reporting of OIs across all economic sectors in Sweden was estimated to be approximately 50%.^4^ In a recent study published by Orellana *et al*, 27% of OIs in Sweden in 2013 were not captured in the official injury register.^5^ This estimate was restricted to the public sector and to private companies with more than 50 employees, thus is a conservative estimate of the overall under-reporting as smaller privately held companies have lower reporting rates.^6 7^ Precarious employment (PE), which is characterised by employment insecurity, income inadequacy and lack of rights and protection, is a well-known social determinant of health and health inequalities and has been associated in the last decades with several adverse mental and physical health outcomes in workers.^8 9^ Several studies have indicated a positive association between certain dimensions of PE and OIs.^10–12^ A systematic review by Koranyi *et al* found that two aspects of PE provided the strongest evidence for an elevated risk of OIs, namely employees working in multiple jobs and being employed by a temporary agency.^10 13 14^ Few studies have directly examined under-reporting of OIs for precariously employed workers. A Canadian study by Shannon and Lowe found differential levels of under-reporting by injury severity, but no associations between under-reporting levels and temporary employment, multiple job holding nor when looking at occupation.^1^ Probst *et al* found in a comparative study between USA and Italy that the tendency to under-report workplace injuries increased with increased perception of job insecurity.^15^ Due to their insecure position, precariously employed workers face more complex decisions whether to report OIs and may accept certain injury hazards as the price of employment.^15 16^ Rich evidence from qualitative studies shows that precariously employed workers identify fear of employment reprisal as one of the main reasons for accepting unsafe conditions and OIs.^16–19^ It is also worth noting the tendency towards an enforced work mobility, which suggests that precariously employed workers are likely to have short job tenure, less training about the physical workplace, less acquaintance with other workers and local management, more stressful and heavier work tasks and a higher tendency to work when sick.^9 20^ Not surprisingly they are also likely to be less aware of work hazards since they receive less training and supervision.^21^ Other determinants have been shown to play a role in under-reporting from the employee side such as variation in workers’ experience, loss of over-time work, fear of being labelled as ‘unable to do their job’ or as a ‘complainer’, lack of proper in-job-training or employment insecurity.^2 3 16^ Other explanations for under-reporting of OIs have been identified in the literature at company level, such as poor employer record-keeping practices, lack of knowledge or understanding of the regulations, lack of health and safety procedures, poor organisational safety climate and differential reporting by company size and sector of economic activity.^5 22–24^

To the best of our knowledge, no published study to date has examined the magnitude of under-reporting, specifically among precariously employed workers compared with non-precarious workers, using a multidimensional definition of PE.^15 16^ The lack of accurate data on under-reporting of OIs in the Swedish labour market, and more specifically among precariously employed workers, is a concern to workers, employers, occupational health and safety professionals, unions and policy-makers in order to be able to control hazards and prevent workplace injuries.

### Aim

The aim of this study was to estimate the magnitude of under-reporting of OIs among employed workers in Sweden in 2013 according to level of employment precariousness. Our hypothesis was that under-reporting of OIs is higher among precariously employed workers as compared to non-precarious workers.

### Methods

#### Data sources

This is a register-based study of OI among individuals aged 18–65 years old residing and working in Sweden in 2013. Data were extracted from four registers, which are described in greater detail in our published protocol^25^: (1) the Longitudinal Integration Database for Health Insurance and Labour Market Studies register (LISA) for employment and demographic data and for development of a PE score; (2) the Information System on Occupational Injuries (ISA), a national OIs register held by the Swedish Work Environment Authority; (3) the AFA, a mutual insurance company is owned by employers’ organisations and trade unions and provides a separate source of OIs reports and (4) the National Patient Register (NPR) to characterise injury severity.

Exclusion criteria were: (1) incomplete information for measuring the exposure variable (PE level), (2) death, emigration or immigration during the year, (3) OIs occurred during transit to/from work since they are reported to traffic insurance instead, (4) injuries due to accumulated exposure and near injuries were also excluded from this study since they are not included in the definition of OIs required to be reported and (5)<90% probability that the employer of the individual paid occupational pension. This last criterion is because one of the two data sources for injury reports only includes employers that pay insurance fees, which is essentially equivalent to paying occupational pensions. Consequently, all self-employed persons and persons working for small companies (<10 employees) were excluded. See flow chart of the total population in online supplemental figure S1.

10.1136/oemed-2021-107856.supp1 Supplementary data

### Employment and demographic data

Sociodemographic and employment data were collected for the year 2013 from LISA providing individual-level data on sex, age, country of birth, highest completed education, family composition and occupation. Data on individuals’ employers were also collected from LISA in order to construct a PE score, including reference employer (largest source of income in November) as well as secondary and tertiary employers, economic sector, number of employees in the company and ownership sector. Reference employer was also retrieved for year 2011 and 2012. We adapted the Jonsson *et al* PE score that was based on a total of five items within three dimensions: employment insecurity, income inadequacy and lack of rights and protection.^26^ For the purpose of this study, only employment insecurity and income inadequacy were included in the PE score. Lack of rights and protection could not be included directly given the unavailability of the data. Employment scores were categorised in three groups resulting in a score ranging between −7 and +2. The PE score was then categorised as being precarious (−7 to −3), borderline precarious (−2 to −1) and non-precarious (0–2).

### Occupational Injuries

Information on all reported non-fatal OIs were retrieved from two data sources: the ISA register and the AFA Insurance records. Both ISA and AFA use the Swedish Legal definition of OIs: ‘an OI is an injury due to accident(s), which occurred at the workplace or other place where the injured person had been for work. For an event to be counted as an accident, it is required that the course was relatively short and arose in connection with a particular event’.^27^

Official ISA statistics covers all employees in Sweden and the employee is responsible for notifying the employer of the OI who is obligated to report it. The AFA register primarily includes workplaces that are covered by a collective bargaining agreement. Employees report directly into AFA through an online form.

### Injury severity

Injury severity was operationalised using data from the NPR, which includes all visits to inpatient or specialised outpatient care. OIs in ISA and AFA were linked based on a ±7 days range, considering injuries reported within a week in either of them as being the same workplace injury. Similarly, information on severity was added from the NPR linking date of admission with injury date on a on a ±7 days’ range. Finally, OIs severity was operationalised following three levels of increasing OIs severity: no healthcare (no admission in NPR), outpatient care and hospitalisation. Individuals were linked across years with the use of an (anonymised) identification number replacing the unique Swedish personal identification number.

### Statistical analysis

We applied a two-source capture–recapture method, estimating the total number of OIs, including those not reported to either source, using the Lincoln-Peterson estimator that assumes source independence.^28^ Ascertainment for each data source was calculated as the actual number of OIs divided by the capture–recapture estimate. Estimates were computed separately for OI severity, all sociodemographic characteristics, occupations and PE levels. To adjust for predictors (sex, age and country of birth) and make the independence assumption more plausible, we calculated our estimates by means of log-linear regression models.^29^ Data management was conducted using SAS V.9.4. Capture–recapture estimates were obtained in R (R Foundation for Statistical Computing), including bootstrap to obtain 95% CIs.

### Results

After merging datasets from ISA and AFA (70 063 and 44 075 injuries, respectively), a final sample for analysis included 82 949 unique OIs, of which 31 189 (37.6%) overlapped. The capture–recapture analysis resulted in a mean estimate of 13 522 under-reported OIs. The distribution of reported OIs and estimates of under-reporting by sociodemographic factors, severity and level of precariousness are presented in table 1. Under-reporting of OIs was 50% higher (22.6/15=1.50) among precariously employed workers as compared with those standardly employed and it followed a dose–response pattern according to the levels of precariousness (the more the precarious, the more the under-reporting) as evidenced by the precarious (22.6%, 95% CI 21.3% to 23.8%), followed by borderline precarious (17.6%, 95% CI 17.1% to 18.2%) and lastly the non-precarious (15.0%, 95% CI 14.7% to 15.3%).

**Table 1:** Capture–recapture estimates of under-reported non-fatal occupational injuries by sociodemographic factors, severity and employment relationship in 2013 in Sweden

	Precarious (7.6%)		Borderline precarious (27.9%)		Non-precarious (64.5%)	
	Total observed	Under-report % (95% CI)	Total observed	Under-report % (95% CI)	Total observed	Under-report % (95% CI)
Total	6275	22.6 (21.3 to 23.8)	23 179	17.6 (17.1 to 18.2)	53 495	15.0 (14.7 to 15.3)
Gender						
Male	2757	21.6 (20.1 to 23.2)	9594	16.4 (15.6 to 17.1)	28 940	13.2 (12.8 to 13.6)
Female	3518	22.9 (21.3 to 24.5)	13 585	18.0 (17.2 to 18.7)	24 555	17.1 (16.5 to 17.6)
Age						
18–24	1746	24.0 (21.6 to 26.3)	2038	19.8 (17.9 to 21.8)	2231	16.9 (15.3 to 18.5)
25–34	1916	23.3 (21.1 to 25.5)	5338	19.0 (17.8 to 20.1)	9812	14.3 (13.5 to 15)
35–54	2015	21.2 (19.2 to 23.2)	10 803	17.1 (16.3 to 17.9)	28 453	15.2 (14.7 to 15.6)
55–65	598	20.6 (17.1 to 24.1)	5000	16.6 (15.4 to 17.7)	12 999	14.8 (14.2 to 15.4)
Country of birth						
Sweden	5224	22.3 (21.0 to 23.7)	18 801	17.6 (17.0 to 18.2)	44 090	14.7 (14.4 to 15.1)
Nordic countries	105	24.6 (14.5 to 34.7)	544	16.4 (13.0 to 19.7)	1449	14.2 (12.4 to 16.0)
Europe	370	21.9 (17.4 to 26.5)	1505	17.5 (15.5 to 19.5)	3674	16.0 (14.8 to 17.3)
Non-Europe	552	24.3 (20.6 to 27.9)	2225	17.9 (16.1 to 19.7)	4062	17.5 (16.2 to 18.8)
Missing	348	–	–	–	–	–
Highest educational level						
Primary school	640	20.4 (17.2 to 23.6)	2915	16.5 (15.2 to 17.9)	6114	13.3 (12.4 to 14.2)
Secondary school	4089	22.0 (20.6 to 23.4)	14 630	17.0 (16.4 to 17.7)	32 099	15.5 (15.1 to 15.9)
Tertiary education <3 years	847	23.8 (20.7 to 26.9)	2610	17.9 (16.3 to 19.5)	7505	10.2 (9.5 to 10.9)
Tertiary education ≥3 years	690	26.1 (21.9 to 30.3)	2957	21.7 (19.8 to 23.6)	7653	20.2 (19.1 to 21.3)
Family composition						
Single	2689	23.3 (21.6 to 25.0)	7662	17.4 (16.5 to 18.3)	17 688	14.6 (14.0 to 15.2)
Single with children	768	21.5 (18.1 to 25.0)	2374	19.7 (18.0 to 21.5)	5174	16.9 (15.7 to 18.0)
Couple with children	2274	23.2 (21.3 to 25.1)	9584	17.8 (17.0 to 18.7)	22 079	15.0 (14.5 to 15.5)
Couple with no children	544	18.0 (14.3 to 21.6)	3559	16.2 (14.8 to 17.6)	8554	14.7 (13.9 to 15.5)

Nordic countries: Denmark, Finland, Iceland, Norway, without Sweden.

Europe: European continent including member and non-members of the EU-28 countries (without Nordic countries)

We did not find differential under-reporting associated with country of birth or family composition (table 1). Non-Swedish born individuals were further merged in one unique group in order to increase statistical power, but results were once again non-significant. Under-reporting was higher among females compared with males (17.8%, 95% CI 17.4% to 18.3% vs 14.4%, 95% CI 14.0% to 14.7%), younger compared with older (19.8%, 95% CI 18.6% to 21.0% vs 15.5%, 95% CI 14.9% to 16.0%) (online supplemental S2). Notably under-reporting increased with increased educational level and this pattern was consistent across all levels of precariousness (table 1).

Under-reporting estimates by injury severity are presented in table 2. Under-reporting of the OIs decreased as the injury severity increased and increased with increasing precariousness across all severity groups. As an example, injuries for which the workers did not seek healthcare, under-report estimates were higher among the precarious 25.6 (95% CI 24.1 to 27.0), followed by the borderline precarious 19.7 (95% CI 19.0 to 20.3) and non-precarious 16.8 (95% CI 16.4 to 17.2). While for OIs resulting in outpatient visits and hospitalisation the captured proportions were similar in both ISA and AFA across all employment relationship groups, the proportions of OIs for which workers that did not seek healthcare were notably higher in ISA (~60%–70%) than AFA (~32%–40%).

**Table 2:** Capture–recapture estimates of under-reported non-fatal occupational injuries (n=82 949) by severity and employment relationship in 2013 in Sweden

Injury severity	Total observed	Under-report % (95% CI)	Captured ISA % (95% CI)	Captured AFA % (95% CI)
Total				
No healthcare	67 739	18.1 (17.8 to 18.5)	70.2 (69.7 to 70.6)	39.2 (38.9 to 39.6)
Outpatient	13 494	8.9 (8.5 to 9.4)	71.5 (70.7 to 72.4)	68.4 (67.6 to 69.3)
Hospitalised	1716	3.4 (2.9 to 4.0)	78.9 (77.0 to 80.9)	83.6 (81.7 to 85.5)
Precarious				
No healthcare	5021	25.6 (24.1 to 27.0)	61.9 (60.2 to 63.6)	32.9 (31.7 to 34.1)
Outpatient	1139	12.8 (11.0 to 14.5)	66.7 (63.7 to 69.7)	61.7 (58.7 to 64.6)
Hospitalised	115	7.3 (3.6 to 11.0)	67.7 (59.1 to 76.3)	77.4 (69.1 to 85.7)
Borderline precarious				
No healthcare	18 986	19.7 (19.0 to 20.3)	68.4 (67.6 to 69.2)	37.7 (37.0 to 38.3)
Outpatient	3684	9.9 (9.0 to 10.7)	69.8 (68.2 to 71.4)	67.4 (65.8 to 69.1)
Hospitalised	509	4.2 (3.0 to 5.4)	78.0 (74.1 to 81.8)	81.2 (77.4 to 84.9)
Non-precarious				
No healthcare	43 732	16.8 (16.4 to 17.2)	71.7 (71.2 to 72.3)	40.6 (40.1 to 41.0)
Outpatient	8671	8.2 (7.8 to 8.7)	72.8 (71.8 to 73.9)	69.7 (68.7 to 70.7)
Hospitalised	1092	2.9 (2.2 to 3.5)	80.5 (78.0 to 83.0)	85.3 (83.0 to 87.6)

AFA, AFA Insurance; ISA, Information System on Occupational Injuries.

Table 3 presents the estimates of under-reporting for the 15 occupations with highest number of reported OIs in 2013, stratified by employment relationship. These occupations represent 91% of the total occupations with reported work injuries that year. While not all the findings were statistically significant across the occupations, we observed higher under-reporting in the precarious and borderline precarious groups as compared with the non-precarious group. The numbers of reported OIs in the precarious group are small, as reflected in the width of the CIs. Nevertheless, in the case of the personal and protectives services, extraction and building trades, sales and services elementary occupations, and models, salespersons and demonstrators, a higher under-reporting was found among the precarious compared with the other groups.

**Table 3:** Under-reporting estimates of non-fatal occupational injuries (n=82 949) in 2013 in Sweden among occupations reporting highest no of injuries (91% of total no of injuries) and sorted by highest under-reporting in the very precarious group

	Precarious		Borderline precarious		Non-precarious	
**Occupations**	**Total observed**	**Under-report %** (**95% CI**)	**Total observed**	**Under-report %** (**95% CI**)	**Total observed**	**Under-report %** (**95% CI**)
Teaching professionals	131	33.2 (23.0 to 43.3)	440	21.5 (16.9 to 26.0)	1293	21.7 (19.2 to 24.3)
Physical and engineering science associate professionals	67	28.0 (15.3 to 40.0)	428	19.3 (15 to 23.5)	1276	15.2 (13.3 to 17.2)
Models, salespersons and demonstrators	383	26.9 (22.0 to 31.0)	805	22.8 (19.7 to 25.8)	961	19.0 (16.5 to 21.4)
Life science and health associate professionals	176	26.7 (16.2 to 37.1)	790	20.7 (16.8 to 24.7)	2090	20.3 (17.7 to 22.8)
Sales and services elementary occupations	556	26.1 (22.2 to 30.1)	2180	17.5 (15.9 to 19.2)	1753	15.4 (13.7 to 17.2)
Extraction and building trades workers	405	23.3 (19.1 to 27.5)	1888	14.9 (13.4 to 16.4)	5103	14.8 (13.9 to 15.8)
Personal and protective services workers	2025	21.6 (19.3 to 23.8)	8052	16.6 (15.7 to 17.6)	12 491	16.8 (16.0 to 17.6)
Other associate professionals	233	21.1 (15.1 to 27.1)	786	14.3 (11.9 to 16.7)	3646	5.9 (5.3 to 6.6)
Drivers and mobile-plant operators	328	19.4 (14.9 to 23.8)	1157	16.2 (14.0 to 18.4)	2572	13.2 (11.9 to 14.5)
Other professionals	75	19.0 (9.2 to 28.8)	403	24.9 (19.9 to 29.9)	1243	21.6 (18.8 to 24.4)
Metal, machinery and related trades workers	215	18.6 (13.2 to 23.9)	967	14.8 (12.6 to 17.0)	3237	12.8 (11.6 to 14.0)
Office clerks	338	18.6 (14.0 to 23.1)	994	14.9 (12.6 to 17.2)	2423	15.4 (13.8 to 16.9)
Teaching associate professionals	109	16.3 (8.9 to 23.6)	541	18.4 (15.0 to 21.8)	1381	19.2 (17.1 to 21.3)
Machine operators and assemblers	438	15.7 (12.0 to 19.5)	1452	14.3 (12.5 to 16.2)	5989	13.5 (12.5 to 14.4)
Stationary plant and related operators	64	6.9 (1.5 to 10.2)	433	6.8 (4.7 to 8.8)	2764	6.9 (6.0 to 7.7)

Finally, under-reporting was examined separately for each of the PE dimensions (employment insecurity and income inadequacy) that had been combined to create the PE score. No differences in the under-reporting of OIs were found according to contractual relation insecurity. Within the employment insecurity dimension, under-reporting was higher in workers holding an unstable position (20.0%, 95% CI 19.3% to 20.7%) as compared with those with a stable one (15.4%, 95% CI 15.1% to 15.7%). Similarly, those in multiple jobs (three or more employers) presented higher under-reporting of OIs (21.4%, 95% CI 20.0% to 22.8%) compared with workers holding one or two jobs (16.0%, 95% CI 15.7% to 16.3%) and compared with multiple job holders in multiple sectors (15.9%, 95% CI 13.9% to 17.8%). In the income dimension, higher estimates of under-reporting were found for workers earning >200% of the median and for those earning <60% of the median (table 4). Covariates adjusted models including sex, age and country of birth were also run and crude results were in close agreement with the adjusted results, thus, we present only crude results in the tables. We present the covariate-adjusted model for table 2 in the online supplemental materials as an example. A separate subanalysis was performed aiming at comparing the CBA cut-off of >90% coverage used in this manuscript and the company size cut-off of >50 employees used in a recent published manuscript of our research group, in order to validate results found in both manuscripts.^5^ Results found in this study were in close agreement with those using the company size cut-off, besides the fact that using the company size cut-off of >60 employees resulted in a smaller number of 71 921 injuries (online supplemental S4).

**Table 4:** Under-reporting estimates of non-fatal occupational injuries (n=82 949) by precarious employment dimensions in 2013 in Sweden

		Total observed	Under-reporting % (95% CI)	%Captured ISA (95% CI)	%Captured AFA (95% CI)
**Employment insecurity**	**Contractual relation insecurity**				
	Directly employed	76 036	16.2 (16 to 16.5)	70.8 (70.4 to 71.2)	44.4 (44 to 44.7)
	Agency employed	1054	16.8 (14.4 to 19.3)	70.6 (67 to 74.2)	42.7 (39.8 to 45.5)
	Directly employed and self-employed	5859	15.8 (14.8 to 16.8)	70.4 (68.9 to 71.8)	46.8 (45.5 to 48.1)
	**Contractual temporariness**				
	Stable	67 872	15.4 (15.1 to 15.7)	72.0 (71.6 to 72.4)	44.9 (44.6 to 45.3)
	Unstable	15 077	20.0 (19.3 to 20.7)	65.2 (65.2 to 66.1)	42.6 (41.8 to 43.3)
	**Multiple jobs/sectors**				
	1–2 employer in 1–2 sectors	77 504	16.0 (15.7 to 16.3)	71.0 (70.6 to 71.5)	44.8 (44.4 to 45.1)
	3 or more employers in 1–2 sectors	3951	21.4 (20.0 to 22.8)	64.8 (63.1 to 66.6)	39.2 (37.7 to 40.7)
	3 or more employers in 3+ sectors	1494	15.9 (13.9 to 17.8)	70.7 (67.8 to 73.6)	45.8 (43.4 to 48.1)
**Income inadequacy**	**Income level**				
	>200% of the median	1101	21.9 (19.0 to 24.8)	65.1 (61.5 to 68.8)	37.3 (34.3 to 40.1)
	120%–199% of the median	18 208	14.2 (13.7 to 14.7)	72.4 (71.6 to 73.2)	48.6 (47.9 to 49.4)
	80%–119% of the median	49 704	16.1 (15.7 to 16.4)	70.8 (70.3 to 71.3)	45.0 (44.6 to 45.4)
	60%–79% of the median	10 330	18.7 (17.8 to 19.7)	69.6 (68.4 to 70.9)	38.3 (37.4 to 39.2)
	<60% of the median	3606	21.6 (20.2 to 23.1)	65.8 (63.9 to 67.7)	36.8 (35.4 to 38.3)

AFA, AFA Insurance; ISA, Information System on Occupational Injuries.

### Discussion

Our results support the study hypothesis that under-reporting of OIs is higher among precariously employed workers compared to those in standard employment relationships. Across all sociodemographic characteristics, there is likely to be more under-reporting of OIs among precariously employed workers, also when taking injury severity and occupation into account. Additionally, individual dimensions of PE were associated with under-reporting. Specifically, income, workers with unstable employment and multiple job holders with more than three employers presented the highest under-reporting. Also, our findings stratified by employment conditions suggest that sociodemographic characteristics such as age, gender and immigration are not strongly associated with under-reporting as previously reported by others.^17 19 30 31^

Only a few previous studies exist to which our results can be compared and discussed. Opposite to our results, a small Canadian study found that permanent workers and single job holders were less likely to submit a workers’ compensation claim for OIs compared with temporary and multiple job holding workers.^1^ Data from USA and Italy suggest that perceived job insecurity is associated to higher under-reporting.^15^

Previous studies have associated low injury severity with a higher under-reporting.^1 3^ We expand those findings by showing that under-reporting still is highest among the most precarious groups irrespectively of severity. An unexpected finding was that higher under-reporting was found among both workers earning the least and among those earning the most. This finding is likely interconnected with the increased under-reporting found among individuals with increased educational level. Under-reporting in these two contraposed employment categories may be driven by different reasons: while low educated workers with a low income may tend not to report an OI in order not to lose their job, a highly educated worker with a high income may work in an occupation in which the injury does not impact workability. In some white-collar workplaces the risk of injury might also be perceived (right or wrongly) as so low that the workplace and workers lack awareness and routines for reporting. Furthermore, higher-wage non-precarious workers may not lose earnings while they are off work, which can reduce the incentive to report an OI for workers’ compensation.

It is well known that large, blue-collar, high-risk, male-dominated and highly unionised workplaces, such as the paper and pulp industry, the motor vehicle industry, the police force and firefighters have very strong reporting routines in place in Sweden.^27^ We did not find gender differences although there was a tendency that women had higher under-reporting than men. It has been suggested that male workers have more control over their jobs and receive more safety training than female coworkers,^31^ which could offer some explanation for this finding.

As for gender, estimates of under-reporting were overlapping between age groups except when comparing the youngest to the oldest. The higher under-reporting in the youngest age group may relate to their inexperience, lack of health and safety knowledge and, as for women, lack of proper training.^19^ Other factors potentially explaining a lower OI report among young workers include their willingness to please employers^32^ and the perceived low severity of the injury.^19 33^

Except for the non-precarious strata, we did not find differential under-reporting among immigrants compared with native Swedes. Evidence suggests that regardless of legal status, migrant workers experience several forms of exploitation at work, are less likely to receive workplace health and safety training but more likely to be employed in hazardous work, work longer hours with fewer breaks.^17 30 34^

No previous study has explored under-reporting and occupations in concurrence with worker’s precarious level. Highest overall under-reporting levels were found in female-dominated white-collar professions such as teaching and healthcare, while blue-collar male-dominated occupations in machine and plant operators and assemblers as well as other associate professionals (which is dominated by police). However, there were other occupational groups where under-reporting in the precarious group was higher than in non-precarious group: models, salespersons and demonstrators; sales and services elementary occupations; extraction and building trades workers; personal and protective services workers; and also other associate professionals.

### Strengths and limitations

Our study is strengthened by the use of register data with high coverage and completeness of the working population, allowing a thorough exploration of sociodemographic and occupational characteristics, linking all data sources through the unique personal identity number. Another strength is the possibility to operationalise PE as a multidimensional construct and consequently be able to stratify the working population according to a precarious score. Finally, finding the same definition for OIs in two independent record systems allowed us to employ the capture–recapture methodology to explore under-reporting. Some limitations, however, should be specified. Both self-employed and those not covered by collective bargaining agreements were excluded as they are too unlikely to be covered by AFA insurance to include in the analysis. This may have resulted in having excluded a potential precarious population from our analysis. We define OIs in ISA and AFA as being the same using an overlap of data sources with a ±7 days’ range, leaving the possibility of ISA reported injuries not being the same as injuries accepted for compensation. Also, we cannot differentiate in our results whether it is the employee failing to file the report of the OI to the employer or the employer who fails to report the injury to the register.^22^ Furthermore, when using capture–recapture methodology, methodological issues may arise from the lack of true independence between data sources.^5^ Lastly, the current data are limited by the cross-sectional nature of the research design. Future examination of longitudinal data could explore the importance of cyclical changes in both our exposure and outcome variable.

### Generalisability of results

The present findings suggest that under-reporting of OIs is higher among precariously employed workers in Sweden. We believe that our findings are generalisable to other settings as they are in line with previous studies from other countries. Additionally, this study confirms the existence of under-reporting in specific sections of the workforce well known in the international literature for their labour market vulnerability—young workers, women and migrant workers. Nevertheless, our results are not generalisable to self-employed workers as well as workers employed in small companies, which were excluded in this study and may present additional and unknown mechanisms on top of those identified here.

### Implications

Even though the present findings have important implications for both employers and employees, any financial, health and social consequence or responsibility arising from the injury shifts from the employer to the employee for each workplace injury not reported. Such consequences may particularly burden precariously employed workers who may not access social benefits for which they qualify through their employment. In addition, OIs if not properly treated could worsen and cause even greater consequences in the long term, as well as contributing to presenteeism and productivity loss.^35 36^ On the other hand, employers may experience losses in wages and productivity, as well as damage to the organisations’ reputation and capacity of recruitment and retention of workers.^37^ By increasing the understanding between PE and under-reporting of OIs, organisations may learn better approaches to improve reporting, address root causes of workplace injuries, and design health and safety programmes aimed at tackling specific component of the workforce—for example, precariously employed workers. Therefore, a good reporting system in the workplace is needed to assure reliable data so that effective and targeted educational, regulatory and technological interventions can be implemented.

### Conclusions

To the best of our knowledge, this is the first register-based study to empirically demonstrate that under-reporting of OIs is consistently higher among precariously employed workers in Sweden. Under-reporting of injuries poses a major problem when it comes to the surveillance of OIs and targeted preventive measures. This problem is compounded when occurring among workers that are most vulnerable in the labour market—precariously employed workers.

## Data Availability

Data can be obtained through acquisition from Swedish registers. The data collection process is described in the method section of this article. Details from the analysis can be obtained from the corresponding author on request.
