# Supplementary material for: Under-reporting of non-fatal occupational injuries among precarious and non-precarious workers in Sweden
Source: Occup Environ Med. 2021 Sep 20;79(1):3–9. doi: 10.1136/oemed-2021-107856 (PMC8685629; doi:10.1136/oemed-2021-107856)
Supplement: Supplementary data [file oemed-2021-107856supp001.pdf]

Supplementary Material 1. Flow chart total population included in the study in 2013.

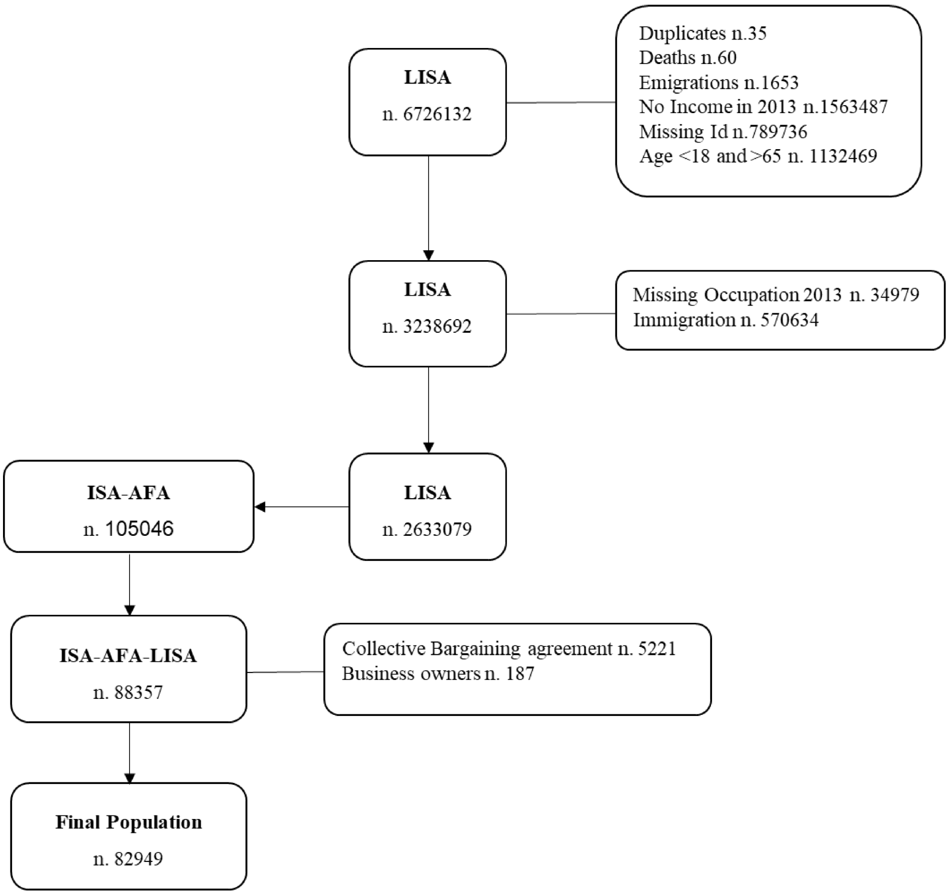

Supplementary material 2. Capture–recapture estimates of under-reported non-fatal occupational injuries by socio-demographic factors in 2013 in Sweden.

	Total observed*	Under-report % (95% CI)
**Gender**		
male	41291	14.4 (14-14.7)
female	41658	17.8 (17.4-18.3)
**Age**		
18-24	6015	19.8 (18.6-21)
25-34	17066	16.5 (15.9-17.1)
35-54	41271	15.9 (15.5-16.3)
55-65	18597	15.5 (14.9-16)
**Country of birth**		
Sweden	68115	16.0 (15.7-16.3)
Nordic countries	2098	15.1 (13.5-16.8)
EU-28	5549	16.8 (15.8-17.8)
Non EU-28	6839	18.2 (17.2-19.2)
**Educational Level**		
Primary school	9669	14.7 (14-15.4)
Secondary school	50818	16.4 (16-16.7)
Tertiary education <3y	10962	12.6 (12-13.3)
Tertiary education >=3y	11300	20.9 (20-21.9)
**Family composition**		
single	28039	16.1 (15.6-16.5)
single with children	8316	18.1 (17.1-19.0)
couple with children	33937	16.3 (15.9-16.7)
couple with no children	12657	15.3 (14.6-15.9)

*Total observed is the total of those injuries reported to ISA only, AFA only and their overlap.

Supplementary Material 3. Capture–recapture estimates of under-reported non-fatal occupational injuries by severity and employment relationship in 2013 in Sweden adjusted by sex, age and country of birth.

**Injury Severity**	Total observed	Underreport % (95% CI)	Captured ISA % (95% CI)	Captured AFA % (95% CI)
**Total**				
no healthcare	67441	18.0 (17.7-18.3)	70.3 (69.8-70.7)	39.3 (39.0-39.7)
outpatient	13448	9.04 (8.63-9.46)	71.5 (70.7-72.3)	68.4 (67.6-69.39
hospitalized	1712	3.6 (2.97-4.24)	78.8 (76.8-80.8)	83.5 (81.5-85.4)
**Precarious**				
no healthcare	5001	25.4 (23.9-26.8)	62.1 (60.3-63.8)	33.0 (31.8-34.2)
outpatient	1135	12.9 (11.1-14.7)	66.7 (63.6-69.7)	61.6 (58.7-64.6)
hospitalized	115	7.75 (3.26-12.2)	67.5 (58.8-76.2)	77.2 (68.7-85.7)
**Borderline Precarious**				
no healthcare	18902	19.4 (18.7-20.0)	68.7 (67.9-69.6)	37.8 (37.2-38.5)
outpatient	3665	9.86 (9.03-10.7)	69.8 (68.2-71.4)	67.5 (65.9-69.1)
hospitalized	508	4.09 (2.82-5.37)	78.0 (74.3-81.8)	81.2 (77.5-84.9)
**Non-Precarious**				
no healthcare	43538	16.7 (16.3-17.1)	71.8 (71.3-72.4)	40.7 (40.2-41.2)
outpatient	8648	8.26 (7.76-8.75)	72.8 (71.7-73.9)	69.7 (68.7-70.8)
hospitalized	1089	2.96 (2.26-3.65)	80.4 (77.9-82.9)	85.2 (82.9-87.5)

AFA= AFA Insurance
ISA= the Information System on occupational injuries.

Supplementary material 4. Capture–recapture estimates of under-reported non-fatal occupational injuries by severity and employment relationship in 2013 in Sweden using company size cut-off of ≥50 employees as Orellana et al (2020).

	Total observed	Underreport % (95% CI)	Captured ISA % (95% CI)	Captured AFA % (95% CI)
**Total**				
no healthcare	62319	16.6 (16.3-17.0)	72.7 (72.3-73.2)	39.0 (38.6-39.3)
outpatient	10809	7.95 (7.54-8.35)	74.4 (73.5-75.3)	69.0 (68.1-69.9)
hospitalized	1236	3.56 (2.85-4.26)	80.0 (77.5-82.5)	82.2 (80.0-84.5)
**Precarious**				
no healthcare	4364	22.8 (21.3-24.2)	66 (64.2-67.8)	32.9 (31.6-34.2)
outpatient	843	11.2 (9.25-13.1)	70.1 (66.7-73.5)	62.7 (59.3-66.1)
hospitalized	69	8 (1.99-14)	66.7 (54.7-78.6)	76 (63.9-88.1)
**Borderline Precarious**				
no healthcare	17303	17.9 (17.2-18.6)	71.4 (70.5-72.3)	37.4 (36.7-38.1)
outpatient	2839	8.7 (7.85-9.54)	73 (71.2-74.7)	67.8 (66-69.6)
hospitalized	358	4.52 (2.97-6.07)	77.1 (72.3-81.9)	80.3 (75.9-84.6)
**Non-Precarious**				
no healthcare	40652	15.6 (15.2-16)	73.9 (73.3-74.5)	40.3 (39.8-40.8)
outpatient	7127	7.34 (6.87-7.82)	75.4 (74.3-76.5)	70.2 (69.1-71.3)
hospitalized	809	2.91 (2.16-3.66)	82.3 (79.585.2)	83.5 (80.7-86.3)

AFA= AFA Insurance
ISA= the Information System on occupational injuries.
